# How context changes the neural basis of perception and language

**DOI:** 10.1016/j.isci.2021.102392

**Published:** 2021-04-02

**Authors:** Roel M. Willems, Marius V. Peelen

**Affiliations:** 1 Donders Institute for Brain, Cognition and Behaviour, Radboud University, Nijmegen, the Netherlands; 2 Centre for Language Studies, Radboud University, Nijmegen, the Netherlands; 3 Max Planck Institute for Psycholinguistics, Nijmegen, the Netherlands

**Keywords:** Biological Sciences, Cognitive Neuroscience, Neuroscience, Sensory Neuroscience

## Abstract

Cognitive processes—from basic sensory analysis to language understanding—are typically contextualized. While the importance of considering context for understanding cognition has long been recognized in psychology and philosophy, it has not yet had much impact on cognitive neuroscience research, where cognition is often studied in decontextualized paradigms. Here, we present examples of recent studies showing that context changes the neural basis of diverse cognitive processes, including perception, attention, memory, and language. Within the domains of perception and language, we review neuroimaging results showing that context interacts with stimulus processing, changes activity in classical perception and language regions, and recruits additional brain regions that contribute crucially to naturalistic perception and language. We discuss how contextualized cognitive neuroscience will allow for discovering new principles of the mind and brain.

## Highlights

• The effect of context is pervasive and present at multiple levels of processing
• Context both facilitates and changes cognitive processing
• Context can be incorporated and manipulated in tightly controlled experiments
• Contextualizing cognitive neuroscience research increases its relevance

## Graphical abstract

**Figure:**
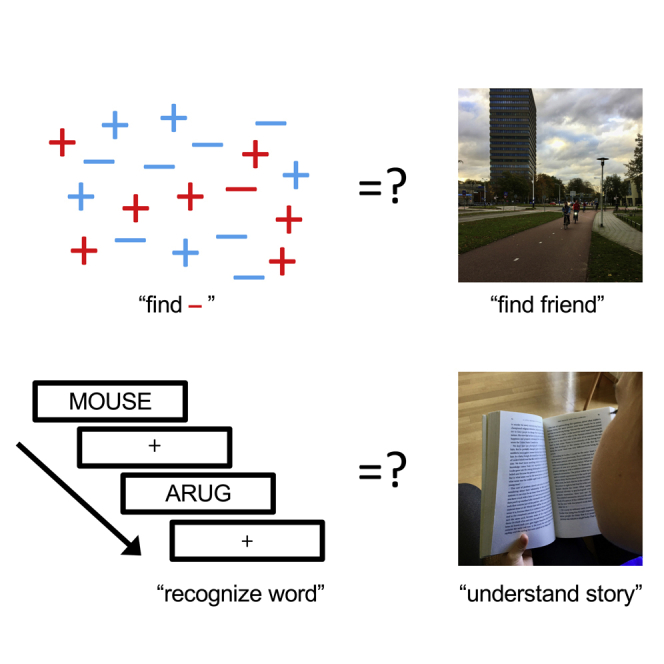

## Introduction

Observing two people shouting at each other would usually evoke a strong emotional reaction in the observer; this depends however on the context in which the event takes place: observing the same social interaction in the context of a film shoot may evoke a very different reaction. The effect of context is pervasive and present at multiple levels of processing—from visual perception to language. For example, a distant object will be perceived as a boat when situated on a lake but as a car when situated on a road; and the sentence “sometimes it is better to talk less” can be a general advice in a presentation course or a request to shut up in a meeting.

In addition to qualitatively changing cognitive processing, context also facilitates processing, allowing us to rapidly understand complex scenes and events. For example, context allows for predicting what someone will do next, facilitating the processing of that action when it is executed. The ease with which we move around and get things done in our familiar environments attests to how well suited our brains are to navigate seemingly complex cognitive spaces. Clearly, the brain is well adapted and intricately coupled to its rich natural habitat. Because of the strong influence of context, we argue that cognitive neuroscience needs to be (re-)contextualized: considering natural context should be the starting point of cognitive neuroscience research.

**Figure 1:**
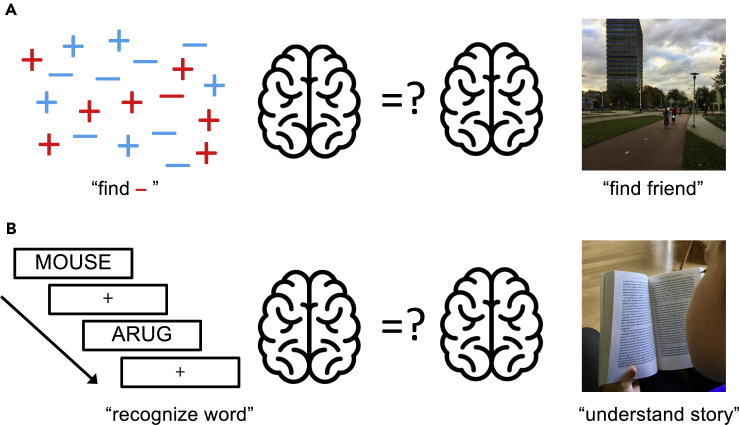
Contextualizing cognitive neuroscience Can traditional research stimuli be used to understand contextualized cognition? (A) Artificial visual search displays (upper left) are often used to study the neural basis of visual attention (“find the red –”). It is unclear, however, if or how neural mechanisms involved in artificial visual search are similarly implicated in searching for objects in natural scenes (e.g. bicyclists in the upper right). (B) The lexical decision task (lower left) is used to study the neural basis of language understanding. The link with language understanding in the real world (e.g. when reading a book, lower right) is not clear. The crucial question is whether the brain processes investigated with decontextualized paradigms (left) can be translated to contextualized settings (right). In the paper, we argue that the cognitive and neural processes involved in contextualized cognition can be qualitatively different than those in decontextualized cognition. Brain icon from Flaticon.com

When browsing articles in cognitive neuroscience journals, it becomes clear that this is rarely done. Instead, the focus is on understanding the neural mechanisms of highly artificial and decontextualized cognitive tasks, such as detecting oriented gratings in rapid visual streams, memorizing multiple simple stimuli across brief delays, and speeded reading of letter sequences (Figure 1). The contrast with cognition as it operates in real life is stark. In real life, we perceive, read, think, and act in rich familiar environments, set within a particular time, place, and culture.

These considerations raise the question of how well cognition can be approximated by decontextualized laboratory tasks. Do the cognitive processes isolated by these tasks operate similarly in the real world? Is it valid to reduce complex behavior to a collection of isolated components? Can we study cognition without considering context? Some of these issues have featured prominently in psychology and philosophy (Clark, 1997; Gibson, 1979; Hutchins, 1996; Neisser, 1976; Varela et al., 1991), but they have not yet significantly influenced the field of cognitive neuroscience.

What do we mean by context? According to dictionaries, context is “The situation within which something exists or happens, and that can help explain it” (Cambridge Dictionary), or “The interrelated conditions in which something exists or occurs” (the Merriam-Webster Dictionary). Although these definitions are admittedly broad, they help point out how context implies situatedness. Context can relate to situatedness at different spatial and temporal scales. For example, context can be of short or long duration, forms that are unlikely to be comparable: the context of a social setting is not comparable to the context of a personal lifetime history. Taken to the extreme, one may argue that the specific context in which a cognitive act takes place is always different, precluding empirical study. In practice however, specific contexts often share structure that makes them generalizable. In this paper, we will show examples of context that have effects that generalize across specific instances and that can thus be studied experimentally.

We now turn to the main argument of the paper: Context can qualitatively change cognitive processing in a way that makes it hard to compare to seemingly similar processes studied in a decontextualized manner. It is common in cognitive neuroscience to consider “context” as an add-on to core cognitive processing. Context is traditionally considered like a Maggi seasoning, “spicing up” the core of cognitive processing. This is unlikely to be correct. Quite on the contrary, context is not something we add on to cognitive operations, it is core to cognition. Without context, cognitive tasks are often difficult and time consuming. With context, many of these tasks become effortless and fast. Context thus both facilitates and changes cognitive processing, as we will see in later examples.

In this paper, we show why incorporating context provides an excellent opportunity for making progress in cognitive neuroscience. We argue that contextualizing research will increase the relevance of research findings for understanding the neural mechanisms of cognition. Relevance is to be understood here in relation to so-called representative design (Brunswik, 1955): Is the research environment representative of the environment the researcher wants to generalize to? For example, findings from research that investigates visual attention in environments that are far removed from real-world environments may not necessarily generalize to real-world conditions and may thus not be optimally relevant for understanding attention.

After brief theoretical considerations, the main part of the paper consists of examples of how taking context into account has changed our views on the neural basis of cognition. Our examples will be focused on visual perception and language since these are our fields of expertise. We believe the paper to be of interest to (cognitive) neuroscientists at large since similar examples could be given regarding other subfields (we refer to a few examples later in the paper). It should be clear that our examples are not meant to be an exhaustive list; they are meant to illustrate our larger point. Finally, in the discussion section, we take stock and describe how our approach relates to other positions. We end the paper by giving recommendations on how contextualization can be used to move cognitive neuroscience forward.

## Relation to the traditional approach

In order to make the case for a contextualized approach, we will first describe how it deviates from the traditional approach to research in cognitive neuroscience. The traditional approach is to isolate small subcomponents of a cognitive process and study those in a decontextualized manner. The overarching rationale of this approach is that the subcomponents can be combined in order to reach understanding of the complete process. In this approach, each subcomponent is a building block, comparable to a piece of Lego that when correctly put together forms a unity, just like separate Lego pieces can form a Lego castle. However, as we will see, context typically interacts with cognitive and neural processing in ways that are not predicted from studying components in isolation, making this assumption unlikely to be correct. This approach has its historical roots in the logic of pure insertion (Donders, 1869). Despite the fact that it has long been recognized that this logic is untenable (Friston et al., 1996; Sternberg, 1969), the traditional approach continues to be the leading paradigm in cognitive neuroscience.

The popularity of the traditional approach is based on the advantage it is believed to have for experimentation: if subcomponents can be studied in isolation, context can be ignored, which makes experimentation easier. An example of how this can be problematic comes from work on the cortical motor system in the macaque monkey. A classical view of the motor cortex is that it is organized somatotopically. This means that each effector (each finger, each hand, each foot, etc.) is controlled by a distinct part of the motor cortex. There is ample evidence for such an organization. Interestingly, however, Graziano and colleagues observed that the classical somatotopy of the motor cortex is variable and state dependent (Graziano and Aflalo, 2007). By varying the timing of cortical stimulation (a factor which was not prominent in earlier work), it was observed that the mapping of actions in the motor cortex does not consist of a neat delineation according to effector but is better characterized by a mapping according to behaviorally relevant actions, like “reach to grasp” or “hand to mouth”. This example shows that the functional organization of the motor cortex will not be correctly understood when mapping each effector separately and assuming that a combination of these mappings together describes the neural basis of complex movements.

A perspective opposed to the traditional approach comes from dynamical systems theory, a general mathematical framework used to describe dynamical systems. In a dynamical system, there is a strong dependence on time for a system to move from being in one state to the other (Chiel and Beer, 1997; Kiverstein and Miller, 2015; Thelen and Smith, 1994; Turkheimer et al., 2021; Ward, 2002). In a dynamical systems framework, it is uninformative to consider components in isolation. The reason for this is that subcomponents of a system are inherently coupled so that they interact in complex, time-dependent nonlinear ways. What is important for the present paper is that when viewed as a dynamical system, it is not informative to investigate neural processing outside of a context since context will have an immediate and complex effect on neural processing. We merely describe the dynamical systems approach as one of several theoretical viewpoints that argue against the traditional building blocks approach to studying cognition. This spirit is also part of enacted and embodied cognition to which we will turn toward the end of the paper.

Now that we have set the stage, we will move to the heart of the paper. We will give examples of how studying cognition within a naturally occurring context leads to a different understanding of the brain basis of cognition as compared to when studying it outside of a context.

## Context in perception, attention, and memory

### Object perception

**Figure 2:**
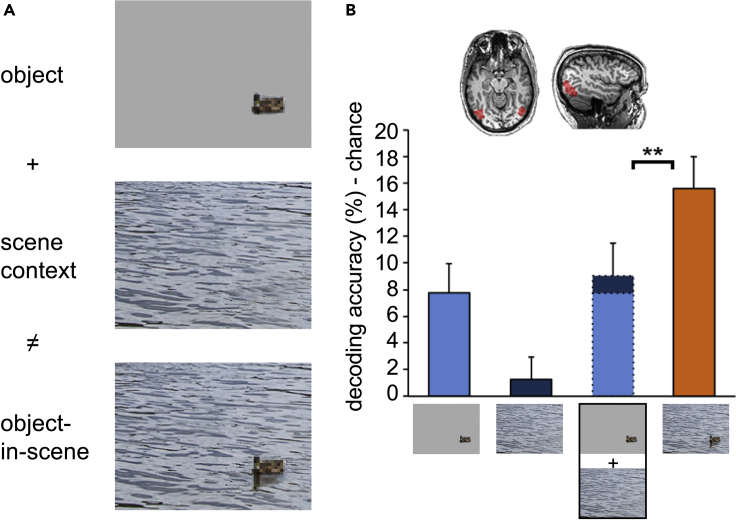
Context effects in object perception (A) An example of a poorly visible (degraded) object that is unrecognizable when presented outside of scene context (top picture) but easily recognizable when presented within scene context (bottom picture). (B) fMRI data showing that activity patterns in object-selective visual cortex (region shown in top) provide more information (i.e., better decoding accuracy) about the category of objects when presented within scenes (orange bar) vs outside of scenes (light blue bar). Furthermore, the above chance decoding accuracy for object-in-scene is more than the sum of the decoding accuracies of the object and scene shown separately, indicating an interaction between scene context and object processing. Error bars indicate standard error of the mean. ∗∗*p*<0.01. Figure adapted from Brandman and Peelen (2017) under CC BY 4.0 license.

The dominant approach in the field of object perception is to measure neural responses evoked by individual isolated objects (or object features) presented at central fixation. While these studies are relevant to our understanding of basic visual processing, they miss important contextual contributions to object recognition. Indeed, object processing is influenced by the identity and position of surrounding objects (Quek and Peelen, 2020) as well as the global scene context. For example, objects presented in a coherent scene context (e.g., a car on a road) are more easily recognized than objects presented in an incoherent scene context (e.g., a car on a lake; Bar, 2004; Oliva and Torralba, 2007). This effect can be so strong that objects that would be unrecognizable when viewed in isolation become readily recognizable when viewed in context (Figure 2). These results imply the existence of a context-based route to object recognition, indicating that isolated object processing is only one pathway to object recognition. A recent study showed that functional magnetic resonance imaging (fMRI) and magnetoencephalography activity patterns in the visual cortex evoked by degraded objects in context closely resembled activity patterns evoked by clearly visible objects presented in isolation, despite the lack of object features in the degraded objects (Brandman and Peelen, 2017). These effects could not be explained by additive (parallel) processing of the degraded object and scene when presented separately (Figure 2). This provides evidence for an interaction between simultaneously presented objects and scenes, whereby objects are recognized through the integration of context-based expectations and isolated object features.

### Visual attention

Context is also crucial for attentional selection, for example, during visual search. Visual search is the task of finding relevant objects among irrelevant objects. This is a common task in daily life, for example, when we search for a friend at the airport, for our keys on the table, or for coffee in the supermarket. The neural mechanisms of visual search have mostly been studied using highly simplified displays, typically consisting of arrays of artificial shapes (see Figure 1 for an example). These studies have been fundamentally important for revealing basic neural mechanisms involved in perception and attention. However, later empirical work showed that these studies do not fully explain how the brain rapidly selects familiar objects from complex but meaningful natural scenes (Peelen and Kastner, 2014). One of the reasons for this is that natural scenes give a powerful context that provides information about likely target locations as well as likely target properties, thereby strongly constraining visual search: we start looking for a pen on top of a desk not on the floor (Castelhano and Krzyś, 2020; Oliva and Torralba, 2007; Võ et al., 2019); and we expect objects to be large when nearby and small when far away. Targets are easily missed when these regularities are violated, for example, when the size of targets is inconsistent with the scene context (Eckstein et al., 2017). Recent studies have started to investigate the neural basis of visual search in more naturalistic conditions (e.g. Çukur et al., 2013; Nardo et al., 2011; Peelen and Kastner, 2011; Preston et al., 2013; Summerfield et al., 2006). One of the findings from this research is that attention to high-level object categories (e.g., people) modulates neural responses to natural scenes across the visual field, independently of spatial attention (Peelen et al., 2009). Such spatially global attention effects were previously considered to be unique to low-level features such as orientation, color, or motion direction (Carrasco, 2011). This is an example of how the brain is optimized to perform naturalistic tasks (e.g., detecting people in scenes), efficiently directing attention to complex objects based on high-level object representations and scene context.

### Visual memory

The presence of scene context is equally important for visual memory. Research has shown that we encode and remember only very few details of a natural scene at any given moment, such that even large (but unexpected) changes across views are easily missed (change blindness; Rensink et al., 1997). Despite this objectively poor encoding of scenes, our daily life experience is of a rich, complete, and continuous visual world. This experience may be an illusion, reflecting our ability to access information in the scene as “external memory” at any moment in time (O'Regan, 1992). In the real world, scenes are typically stable and continuously present, such that there is usually no need to encode everything in memory. Rather, the observer only needs to have a coarse representation of the scene to know where relevant information can be found, such that attention and eye movements can be directed to relevant information when needed. Indeed, research has shown that participants make frequent eye movements rather than encode items in working memory when performing naturalistic tasks (Ballard et al., 1995; Draschkow et al., 2021). This implies that the limited capacity of visual working memory (to 3–4 items), a major field of study in cognitive neuroscience, may not often limit performance in daily life (Van der Stigchel, 2020). The neural basis of interactions between internal and external memory remains largely unexplored, opening up interesting research questions with great relevance for understanding memory deficits in neuropsychological disorders and cognitive aging.

### Summary

These examples highlight how visual perception, attention, and memory depend on the presence of naturalistic scene context. Visual, attention, and memory systems have developed and evolved to optimally perform real-world tasks, as also reflected in the remarkable speed of natural scene perception (Li et al., 2002; Potter, 1976; Thorpe et al., 1996). It is thus increasingly appreciated that the brain makes use of a wide range of available information (e.g., scene context, statistical regularities) to support naturalistic perception, attention, and memory (Kaiser et al., 2019). The neural basis of these processes in contextualized settings is still unclear; we expect that much progress can be made in this area in the coming years.

## Context in language comprehension

Decades of cognitive neuroscience research has investigated the neural basis of decontextualized language comprehension, focusing on the understanding of single words and sentences. Naturally, during actual language comprehension, words and sentences can be combined to form larger pieces of discourse such as narratives. In this section, we will show that studying language at the level of decontextualized words and sentences gives an incomplete picture of the neural basis of language. Moreover, context not only extends the brain regions involved in language, it can also alter the neural processes involved in understanding language.

### Context extends the language network in the brain

The traditional neural network for language consists of areas in the middle and anterior temporal cortex, inferior frontal regions, and the inferior parietal lobe (e.g., Binder et al., 2009; Fedorenko and Thompson-Schill, 2014; Hagoort, 2013). Interestingly, once language is processed at the level beyond single sentences, other regions become involved. Examples include information extraction over longer periods of time (e.g., paragraphs within a narrative), which is associated with activation in posterior midline structures such as the precuneus (Jacobs and Willems, 2018; Lerner et al., 2011; Zacks et al., 2010). Another example is the role of anterior medial prefrontal regions thought to be involved in understanding the intentions and beliefs of others (Ferstl et al., 2008; Hagoort and Levinson, 2014; Mason and Just, 2006; Willems and Varley, 2010). In contextualized language comprehension, understanding the intentions of others is so important that it becomes hard to see how the process is not part and parcel of natural language. Put differently, the picture of the language network in the brain changes substantially when contextualized language is studied instead of single words and sentences.

**Figure 3:**
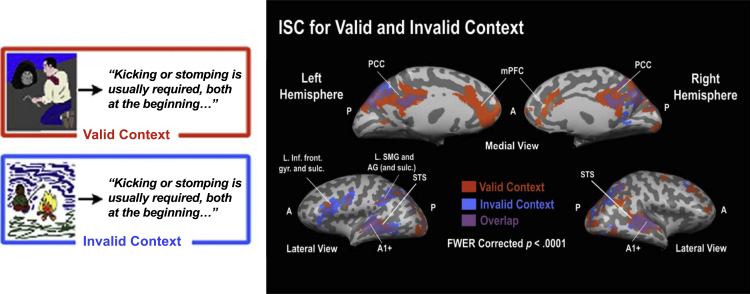
Context effects in language comprehension A text understood within a valid context involves different brain regions than understanding the same text within an invalid context. Participants read a description of an activity, in this example a description of how to change a flat car tire. The description could be preceded by a picture that fitted the description (someone changing a tire; upper left) or not (someone sitting at a fireplace; lower left). The written description is much easier to understand when preceded by a matching picture (valid trials), as compared to when preceded by a non-matching picture. Note that the sentence in the figure was only the start of the description, and the actual stimulus was much longer. Areas of the traditional language network (e.g. middle temporal and inferior frontal regions), as well as areas not traditionally thought to be involved in language comprehension, such as the posterior cingulate cortex (PCC) and anterior medial prefrontal cortex (mPFC), are sensitive to this manipulation, as shown here through analyses of inter-subject similarity of fMRI activity. ISC = inter-subject correlation; A = anterior; P = posterior; A1+ = primary auditory cortex; L = left; SMG = supramarginal gyrus; STS = superior temporal sulcus; AG = angular gyrus. Figure adapted from Ames et al. (2015), with permission from MIT Press Journals.

An example case in point is a study in which discourse coherence was investigated (Ames et al., 2015). Participants read short descriptions (several sentences long) of basic activities (e.g. change a tire on a car; see Figure 3). When the text was preceded by a picture of someone changing a tire, the description was easy to understand. When presented with a different, unrelated picture, the description was hard to understand. It was found that traditional language areas (e.g., middle temporal and inferior frontal areas) were activated in both conditions (Figure 3). The presence or absence of context did not modulate activity in these areas. By contrast, posterior and anterior midline structures (anterior medial prefrontal cortex and posterior cingulate cortex) were more strongly activated when the content of the description could be more easily understood because of the presence of valid context cues (Ames et al., 2015; see also Heidlmayr et al., 2020). These studies show that adding a meaningful context to even a relatively simple piece of language changes the neural areas involved. Some have hypothesized that it is therefore better to either extend the language network to include these additional regions (Ferstl et al., 2008) or to let go of the notion of a language network altogether (Hasson et al., 2018). Either way, these findings make clear that context fundamentally changes the neural basis of language.

### Contextualized grammar

Next to changing the areas involved in language comprehension, context can modulate activation in “core” language areas. Typically, context makes language comprehension easier. An interesting illustration is the context sensitivity of syntactic comprehension (“grammar”), a part of language comprehension considered crucial by many. In an fMRI study, participants were presented with subject-initial sentences (“He noticed her”) and object-initial sentences (“Her, he noticed”; Kristensen et al., 2014). When presented on their own, the object-initial reading is most difficult (both are grammatically correct in Danish). The sentences were presented in two conditions: one absent of context and one where a brief preceding context rendered the (more complex) object-initial sentence more acceptable. For instance, when preceded by the sentence “Peter overlooked all the shoplifters except Anne”, it is the object-initial continuation “Her, he noticed” that is more natural (see also Mak et al., 2008). The authors found that activity in the left inferior frontal gyrus was reduced when context licensed the object-initial continuation, as compared to when the sentences were presented on their own. These results illustrate that findings from single-sentence, out-of-context studies of language do not necessarily extend to findings obtained with language materials that are contextualized, even if the context is rather minimal.

### Context makes language understanding easier

The example of “difficult” sentences (“Her, he noticed”) becoming easy to understand within context is likely to be a general rule. In a compelling conceptual analysis, Van Duijn and colleagues argue that complex multiple mental embeddings that are very difficult to understand outside of a contextual embedding are actually the norm in famous works of fiction (Van Duijn et al., 2015). To illustrate, take the following sentence:

“I *know* that Ann *believes* that Peter *suspects* that Mary *realizes* that Bill *understood* that Will is a sailor.” (Van Duijn et al., 2015)

This sentence shows a mental embedding of 5 degrees. It is very difficult to understand: Try to quickly say what Peter is suspecting about Bill? Knowing who is thinking or believing what about whom is like a puzzle in this example, taking considerable effort. It has been found that areas in the inferior frontal cortex are sensitive to these multiple embeddings (Grodzinsky and Santi, 2008), and it has been argued that this is evidence for the region's functional role in syntax. However, in works of fiction, this kind of embedding is paramount. For example, in Shakespeare's Othello, there are points in the play in which the number of embeddings exceeds five (character 1 thinks of character 2 that she believes that character 3 suggests, etc.; Van Duijn et al., 2015). Spectators not only find such embeddings easy to understand in the context of Shakespeare, they might even be attracted to them, spending time and money to see the play. Therefore, what is difficult outside of context becomes easy to understand and actually enjoyable within a context.

A similar case can be made for (linguistic) perspective switching. Perspective switching (e.g. seeing the world from your own versus from another person's point of view) is cognitively costly, as evidenced by reaction times (e.g. Vukovic and Williams, 2015). However, within a narrative context, perspective shifts occur repeatedly. Moreover, these are not just switches from one perspective to another but are rather complex, multiple person switches occurring within one paragraph, even in ordinary newspaper articles (van Krieken et al., 2019). Details left aside, the point is that context eases the cognitive load of these processes.

This begs the question whether it is justifiable to study processes such as mental embeddings and perspective switching outside of context. What is the relevance of knowing what happens when we read isolated sentences with difficult embedding when we, under normal circumstances, have context to make comprehension so much easier?

We end this section with a note about another core process in language: word comprehension. Many studies have sampled people's reactions to single words, either using brain measures, reaction times, or ratings, to understand how words are processed by the brain (Meteyard and Vigliocco, 2018). On the one hand, insights and ratings gained from these studies correlate with reading measures in more contextualized settings (e.g., lexical frequency during natural reading of narratives; Mak and Willems, 2019). On the other hand, eye movement measures (e.g., gaze duration) are strikingly different when reading the same words within a novel versus within short paragraphs (Dirix et al., 2019). The shared variance between reading under these different circumstances was as low as 1%–14%, depending on the eye movement measure used (Dirix et al., 2019). This finding suggests that the overall context in which words (the same words) are read has a much larger influence on reading times than word characteristics of the words in isolation. Studies that measure responses to words in isolation by definition miss out on these important contextual effects.

### Summary

Language in context is understood and processed differently than decontextualized language. The language network in the brain as established from decontextualized language stimuli is different from the network of areas involved in understanding language at the discourse level (at the level of multi-sentence units such as narratives). Not only do additional brain regions play a role during contextualized language comprehension, it also changes the response of language areas. Phenomena that are difficult to understand without context, such as syntactically complicated sentences, become easy to process once embedded in a context. Taking context seriously will change the way we think about the neural basis of language (Hasson et al., 2018; Willems et al., 2020). It provides ample opportunities to make future research on language and the brain more relevant to understanding this uniquely human capacity.

## Discussion

What are the implications for cognitive neuroscience of taking context seriously? In the remainder, we show how our position can help to guide future experimentation. Our hope is that doing our science differently will increase the relevance of cognitive neuroscience for understanding the workings of the brain in its natural habitat.

### 4E philosophy

Although not mainstream in cognitive neuroscience, thinking of cognition in context has a strong theoretical basis in philosophy of mind. The central tenet of so-called 4E (embedded, embodied, extended, enacted) cognition is that cognition should be thought of as resulting from the interplay between brain, body, and environment. Proponents of 4E cognition consider it a conceptual mistake to understand cognition solely as the processing of internalized models by a brain. Instead, cognition is what happens when the brain and body act in the rich environment that the brain and body are used to act in. As a result, the right unit of analysis is not the brain but the coupled system brain-body-environment (e.g. Chemero, 2009; Newel et al., 2018).

One example of this coupling is cognitive off-loading, in which the environment is used as an external memory (see also the section on visual memory above). The thesis underlying these examples is that of the extended mind, part of 4E cognition. Clark and Chalmers (1998) described “cognitive off-loading” as follows:

“In these cases, the human organism is linked with an external entity in a two-way interaction, creating a *coupled system* that can be seen as a cognitive system in its own right. All the components in the system play an active causal role, and they jointly govern behavior in the same sort of way that cognition usually does.” (Clark and Chalmers, 1998)

Other examples of cognitive off-loading are the use of devices (computers, smartphones) to remember or organize information or to navigate one's environment. Here, we see that cognition is not constrained to the brain and body but is actively extended to the environment (Slors, 2020). Another telling example of the influence of cognitive off-loading comes from research on aging. It is well established that tasks requiring cognitive control and memory decline with age. Interestingly, however, this decline is much less pronounced—or even absent—when older adults are tested in contextualized settings (Phillips et al., 2006). An example of a standard cognitive control task is the Tower of London task (requiring complicated planning), whereas an example of a situated task is buying items in a grocery store (and plan other actions, implemented in multiple errands test; Alderman et al., 2003; Phillips et al., 2006). Indeed, performance on the decontextualized task declines with age, whereas performance on the contextualized task does not. This shows that the older participants can use context to compensate for the apparent decline in cognitive function. The outcome is that they perform better in a contextualized setting than in a setting without context. Decontextualized tasks give an unrealistic impression of how cognitive processes are impacted by aging, making them unsuitable for producing a better insight into these processes.

It is beyond the scope of this paper to go deeper into 4E philosophy. We point interested readers to several excellent contributions (Bruineberg and Rietveld, 2019; Chemero, 2009; Clark, 1997; Gallagher, 2005; Newel et al., 2018; Wheeler, 2005). It is worthwhile to notice that although the 4E movement is mainly conceptual, at least two fields in the broader landscape of cognitive science have benefited greatly from it. Incorporating the idea of environment-agent coupling led to the shift in robotics to situated robotics (Pfeifer and Bongard, 2007). Similarly, animal cognition research has made progress by putting the animal-environment coupling center stage (Barrett, 2011). Cognitive neuroscientists can similarly use this theoretical basis to their advantage.

### Relationship with previous calls for ecological validity

Our call for including context in cognitive neuroscience research is related to recent calls for increasing ecological validity and moving toward “real-world” neuroscience (e.g. Matusz et al., 2019; Nastase et al., 2020; Shamay-Tsoory and Mendelsohn, 2019), which in turn build on older discussions of the topic (e.g. Gibson, 1979; Neisser, 1976). Several methodological advances make ecologically valid research more feasible than in the past, including computational advances (Armeni et al., 2017; Kriegeskorte and Douglas, 2018), virtual reality (Peeters, 2019; Reggente et al., 2018), and the possibility of measuring neural responses in more naturalistic situations (mobile imaging: Parada and Rossi, 2020; bringing objects in the scanner: Snow et al., 2011). We see great promise in these developments because they will allow for including increasingly naturalistic context, allowing researchers to address new questions and discover new principles of the mind and brain. However, our call is not a general call for increasing ecological validity. Rather, we more specifically point to the role of context in cognitive neuroscience. The dogma that taking context into account necessarily leads to a decrease in experiment control is countered by actual experimental practice. As the examples in the previous sections show, context can be incorporated or manipulated in tightly controlled experiments (see also Peeters, 2019).

### Guidelines for future research

What are the implications for cognitive neuroscientists? First and foremost, we want to stress that we do not believe that there is a magic formula for how to “best” do research. What we do suggest is one golden rule that we believe can help our field move forward. The rule is simple yet influential: Define the goal of your research, and make sure that the implementation stays close to that goal. Defining the goal of research is an explicit formulation of the phenomenon that the researcher wants to explain (Willems, 2011). This position is reminiscent of the one formulated by Brunswik (1955), who used the term “representative design” to make the case:

“… representative design means that researchers first need to define a reference class of stimuli, tasks, and situations to which they intend to generalize a result. By sampling from this predefined set of conditions, the set of conditions toward which the generalization is intended is part of the experimental design.” (Brunswik, 1955; discussed in Holleman et al., 2020; see also Nastase et al., 2020).

As a concrete example, if a researcher is interested in syntactic language comprehension during reading, the next step would be to assess how syntactic comprehension naturally takes place. If syntactic comprehension almost never occurs in the form of multiple recursive embeddings (Verhagen, 2009), then it follows that using such recursive language stimuli is not representative of the phenomenon under study since it will not allow generalization to the actual circumstances in which syntactic comprehension takes place (Willems, 2011). Similarly, if one's topic of interest is to study how people find objects in daily life, it is important to consider the role of familiarity and context in visual search, as discussed above.

There is a useful analogy with cognitive neuroscience research done in animals. Animal researchers are often asked to defend why they believe animal results translate to humans. How are insights into spatial cognition in rats informative for how humans navigate? Why does having a neural model of macaque's working memory help us understand how memory works in humans? We argue that, similarly, cognitive neuroscientists need to describe why they believe their experimental design allows for generalization to cognition as it occurs in daily life. Why is it conceivable that the insights gained from a simplified experiment are informative to the part of cognition under study? We believe that being explicit about this link will help cognitive neuroscientists take full potential of the opportunities that a context-centered approach offers and will lead to relevant and translatable insights into the neural basis of cognition.
